# Knowledge, attitudes and practices regarding echinococcosis in Xizang Autonomous Region, China

**DOI:** 10.1186/s12889-020-8314-8

**Published:** 2020-04-15

**Authors:** Nima Qucuo, Guangjian Wu, Ruifeng He, Danzeng Quzhen, Ciren Zhuoga, Suolang Deji, Lijie Zhang, Zhigang Zhao, Zhongjun Du

**Affiliations:** 1 Xizang Autonomous Region Center for Disease Control and Prevention, Lasa, 850000 Xizang Autonomous Region People’s Republic of China; 2 School of Public Health, Jilin University, Changchun, 130021 Jilin Province People’s Republic of China; 3 Shandong Center for Disease Control and Prevention, Jinan, 250014 Shandong Province People’s Republic of China; 4 Academy of Preventive Medicine, Shandong University, Jinan, 250014 Shandong Province People’s Republic of China; 5 Chinese Center For Disease Control And Prevention, Beijing, 100050 Beijing People’s Republic of China; 6 Shandong Academy of Occupational Health and Occupational Medicine, Shandong Academy of Medical Sciences, No. 18877, Jingshi Road, Jinan, Shandong Province 250062 People’s Republic of China

**Keywords:** Echinococcosis, Prevention, Xizang Autonomous Region, China

## Abstract

**Background:**

Echinococcosis is a neglected zoonotic parasitic disease caused echinococcus parasitizes, poseing a significant economic burden on both humans and animals. There are limited studies on echinococcosis in China, especially Xizang Autonomous Region, although the area is endemic area for echinococcosis. The study aimed to provide information for strategic prevention against this disease.

**Methods:**

A cross-sectional survey was conducted among the residents in Xizang Autonomous Region, China to evaluate their knowledge, attitudes and practices on endemicity of echinococcosis. A face-to-face survey was conducted to collect the data using a well-designed questionnaire. The contents included basic personal information, knowledge, attitudes and practices about echinococcosis, personal hygiene and behavior habits, dog feeding and whether they had received the information on echinococcosis, and so on. We surveyed 840 persons in practice. All data analysis was performed using Epi Info 7.2.

**Results:**

Of the total particpants, 86.8% had a primary education level or below (including primary and illiterate), and even 45.0% were illiterate. Farmers and herdsmen represent the main occupations in this study. People who know all the echinococcosis-related knowledge in the questionnaire only accounted for 8.7% of the participants. However, none of the participants was aware of routes of echinococcosis infection in human or dogs. The data showed participants with higher educational background had the high awareness rate of echinococcosis-related knowledge or attitudes (chi-square for trend, χ^2^ = 21.23, *p*<0.05 & χ^2^ = 48.43, *p*<0.05). In addition, The percentage of the participant with awareness of echinococcosis-related practices was associated with their age and principle occupation (χ^2^ = 52.72, *p*<0.05 & χ^2^ = 20.63, *p*<0.05).

**Conclusions:**

Xizang Autonomous Region is an epidemic area of Echinococcosis. The prevalence of the disease has been largely due to the lack of knowledge, awareness, and poor hygiene practice in local residences. Therefore, effective disease prevention education and awareness campaigns in community will be significantly helpful in prevention and control of echinococcosis.

## Background

Echinococcosis is a zoonotic parasitic disease caused by echinococcus parasitizes infection in humans (or animals). It seriously endangers people’s health and life safety, and affects social and economic development [1]. Cysticercosis echinococcosis (CE) and Alveolar echinococcosis (AE) are caused by *E.granulosus* and *E.Multilocularis* infections, respectively [2]. CE is prevalent in most pastoral and range land areas of the world. Highly endemic areas are mainly located in the eastern Mediterranean, Western China, Southern and Eastern Europe, North Africa, the southern part of America, Siberia and Central Asia [3]. AE is prevalent in high latitudes of the Northern Hemisphere countries, such as North America, continental Europe, Siberia, Central Asia, Japan and China [3]. Among them, Gansu, western Sichuan and the Tibetan Plateau in China are the most serious areas of alveolar echinococcosis in the world [4].

In China, AE is also known as “worm cancer”, a fatal and serious disease [5] which is difficult to treat with poor prognosis [6]. After AE infection, the fatality rate of untreated can be large than 90%, even as high as 94% [6, 7]. Echinococcosis not only affects human health and causes human lives to be lost due to significant morbidity and mortality, but also brings about economic loss related to livestock husbandry [8, 9]. The economic loss relating to livestock husbandry can further worsen the accessibility of medical intervention causing a tremendous impact on the health of patients, even with loss of their lives [1]. It is estimated that China accounts for 40% of the world’s 1 million disability-adjusted life years (DALYs) caused by CE, and accounts for 34% of the world’s annual losses of US$ 1.92 billion due to CE. China also held an important share in the annual global livestock production losses associated with CE, reaching US$ 2.92 billion [8]. Previous studies have shown that CE can reduce the productivity of infected animals by 10%, lowering meat quality, milk and fiber production and the number of surviving offspring [10]. The annual number of new cases of AE in China accounts for 91% of the global total of new AE cases, and responsible for 95% of the global DALYs and the corresponding years of life lost (YLLs) due to AE [11].

CE is a chronic, complex disease and defined as a neglected tropical disease (NTD) by the World Health Organization (WHO) [12]. Four options for the treatment of CE are currently available: (1) surgery, (2) chemotherapy with albendazole, mebendazole or other anthelmintic drugs, (3) PAIR (puncture, aspiration, injection, reaspiration), (4) watch and wait for inactive or silent cysts [13]. At present, there is no optimum treatment for CE, and there are no clinical trials to compare the different modalities [14]. Because of the constraints in the health-care system [15], there is no standard for choosing treatment options of CE leaving surgery being still the main method of treatment [16]. Although surgery, as the classic treatment is curative way, it cannot completely prevent recurrence of the disease [14]. In addition, surgery is not suitable for patients with multiple cysts in several organs [2, 14, 17]. With the development and progress of medical technologies, CE can be diagnosed primarily utilizing imaging, immunology, molecular biology and puncture biopsy [13]. In the meantime, a large number of insecticides have been put into use, which has achieved certain results in reducing the incidence of CE [13]. Long-term use of chemical drugs leaves other problems such as drug resistance, drug residues, environmental pollution, and so on [13].

Previous studies have shown that the transmission of echinococcosis is influenced by the economic and cultural conditions of the community [8, 9]. The most important factors are associated with dog, such as ‘close contact with dogs’, ‘home-raising dogs’, ‘feeding dogs with the viscera of diseased animals’, followed by dietary factors, such as ‘lack of washing hands before meals’, “drinking river or stream water” [18–20].

To effectively prevent and control echinococcosis, it is very important to evaluate the level of knowledge about the disease, increase awareness regarding the preventive measures and awareness of risky practices that spread the disease within the community. The objective of this study was to obtain information on the current situation of knowledge, attitudes and practices about echinococcosis among the residents of Xizang Autonomous Region. In addition the aim was also to determine current health promotion activities, to provide helpful information for the improvement and formulation of health education policies for the future.

## Methods

**Fig. 1:**
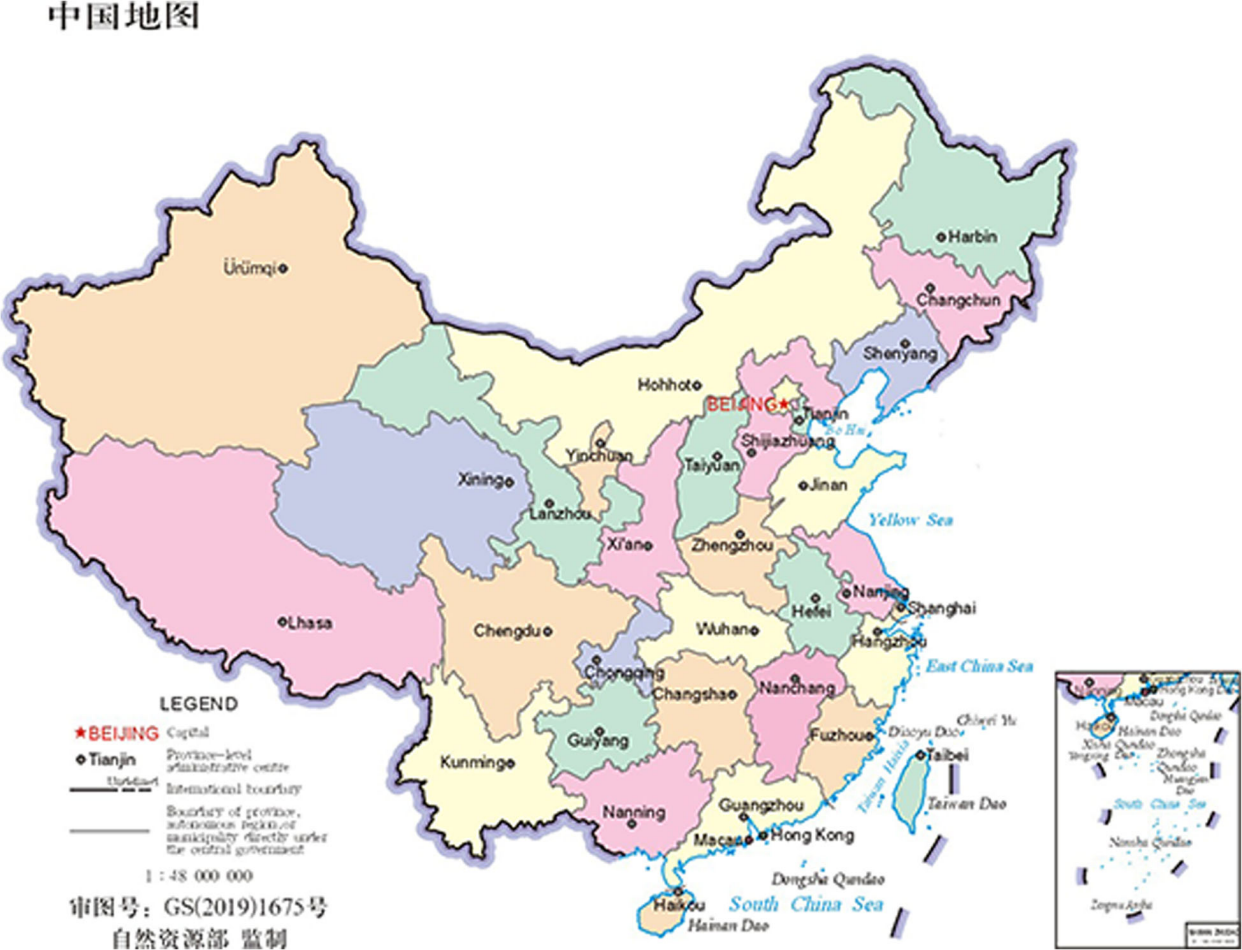
The study area in map of China. (This map is downloaded from the standard map website of the Chinese government and can be used directly. And standard map website: http://bzdt.ch.mnr.gov.cn/browse.html?picId=“4o28b0625501ad13015501ad2bfc0292”. Permissions: The public can browse and download the standard map free of charge from the standard map website, and need to mark the map content approval number when using the standard map directly. The map content approval number we used in this paper is GS (2019)1675, as shown in the figure)

This study was a cross-sectional survey conducted in Xizang Autonomous Region, which locates in the west and south of the Qinghai-Tibet Plateau. It accounts for more than half of the area of the Qinghai-Tibet Plateau. Lhasa is the capital city of Xizang Autonomous Region. The most area of the region is above 4000 m above sea level. As such, Xizang is known as the ‘the roof of the World’ and “the third pole of the earth”, and the highest elevation in the world (Fig. 1).

### Study subjects

The study subjects were permanent residents of Xizang Autonomous Region (Residence time ≥ 6 months).

### Sample size calculation

Calculating sample size was based on simple random sampling.

formula:

$$ n=\frac{u_{\raisebox{1ex}{$a$}\!\left/ \!\raisebox{-1ex}{$2$}\right.}^2\pi \left(1-\pi \right)}{\delta^2} $$

Test significance level was set α = 0.05, admissible error δ = 10%, cognitive eligibility rate was 33% according to the literature. In this study, a multi-stage complex sampling method was used. The design effectiveness was set at 1.5 and the sample size was set at 600 since with a 10% non-response rate, the minimum sample size should be 667.

### Sampling method

Sampling stratification was conducted according to numbers of prefectures and municipalities in Xizang Autonomous Region, and the seven prefectures were divided into seven layers. In each prefecture, three counties (districts) were randomly selected by a simple random sampling method. In each selected counties (districts) two townships and two villages were further randomly selected according to the simple random sampling method. In each selected village selected, 10 local villagers were randomly selected according to the simple random sampling method. Totally, 840 subjects were selected for this study.

### Questionnaire design

The questionnaire (Additional file 1) was developed in Chinese by the Xizang Autonomous Region Center for Disease Control and Prevention based on their past experience and a literature review of comparable studies [21–23]. A series of question were listed with point for each question, and zero for ignorance or mistake. The total scores of knowledge, attitudes and practices of echinococcosis are 15, 6 and 6 respectively. Referring to the “health literacy level of infectious disease prevention and control among Chinese residents” [24], we defined criteria for overall assessment. A total score of 80% or more in each part is classified as high awareness. Therefore, a participant is said to have “high level of awareness” if-- the total score of echinococcosis-knowledge is at least 12 points; if the total score of echinococcosis-attitudes is at least 6 points and total score of echinococcosis-practices is at least 6 points.

### Data analysis

We used Epidata 3.5.1 to double input the data. Participants’ socio-demographic characteristics such as age, gender, education level and principle occupation were presented as frequencies and percentages. The awareness rate of knowledge, attitudes and practices of echinococcosis were calculated as follow:

Rate of echinococcosis knowledge = the number of people who answered a certain knowledge correctly divided by the total number of people being investigated× 100%. Attitude holding rate = the number of people who held a certain attitude divided by the total number of people being investigated× 100%. Practice formation rate = the number of people who formed a certain practice divided by the total number of people being investigated× 100%.

The chi-square test was used to analyse the differences between social demographic characteristics and the awareness rates of relevant indicators. Additionally, the chi-square test for trend was applied to investigate the ordering of awareness rates among the indicators. All data analyses were performed using Epi Info 7.2 (https://www.cdc.gov/epiinfo/pc.html) at 5% level of significant.

## Results

### Socio-demographic characteristics of the study population

The information of socio-demographic characteristics was summarized in in Table 1, Age of the interviewed sujects ranged from 12 to 79 years (mean: 40.9 ± 14.0 standard deviation). The ratio of male to female was 1.3:1. The great majority (86.8%) of the interviewed residents had a primary education level or below, and as many as 45.0% were illiterate. Farmers and herdsmen were the main occupations in this study.

**Table 1:** Socio-demographic characteristics of the residents participated in echinococcosis knowledge, attitudes and practices survey in Xizang Autonomous Region, China (*N* = 840)

Variables	Category	n	%
Age (years)	≤20	38	4.5
	21~30	184	21.9
	31~40	256	30.5
	41~50	145	17.3
	51~60	123	14.6
	61~70	76	9.0
	>70	18	2.1
Gender	Male	478	56.9
	Female	362	43.1
Education level	Illiterate	378	45.0
	Primary	351	41.8
	Secondary	59	7.0
	University	52	6.2
Principle occupation	Farmers	415	49.4
	Herdsmen	274	32.6
	Other	50	6.0
	Cadres or professional technicians	43	5.1
	Workers	30	3.6
	Students	28	3.3

### A lack of echinococcosis-related knowledge among the subjects

#### Awareness rate of echinococcosis-related knowledge

Only 73 persons answered all 15 questions about echinococcosis correctly, accounting for 8.7% (73/840) of the participants. The awareness rates of non-transmission of echinococcosis about human-to-human, human-to-dog and dog-to-dog were all very low. Only 1/4 of the respondents knew that echinococcosis cannot be transmitted by dog-to-dog, were showed in Table 2.

**Table 2:** Awareness rate of echinococcosis-related knowledge among residents of Xizang Autonomous Region, China, 2018 (*N* = 840)

Theme	Variables	Number of awareness	Rate (%)
Routes of human infection	Have you ever heard of echinococcosis?	826	98.3
	Contaminated food by dog manure containing insect eggs can cause disease in humans or livestock	759	90.4
	Contaminated water by dog manure containing insect eggs can cause disease in humans or livestock	745	88.7
	Cannot be transmitted by human-to-human	391	46.5
	Besides dogs, wolves and foxes can also transmit echinococcosis	586	69.8
Routes of dogs infection	Can be transmitted by dog-to-dog	213	25.4
	Cannot be transmitted by human-to-dog	422	50.2
	Dogs are infected by eating the uncooked viscera of sick cattle and sheep	704	83.8
Damage	Damage to human health	793	94.4
	Cause reduction of cattle and sheep production	704	83.8
Prevention	Expelling parasite for dogs	651	77.5
	Cannot feed dogs with uncooked viscera of livestock	722	86.0
	Handle dog manure (be burned or deeply buried)	693	82.5
	Don’t be in close contact with dogs in life (such as kissing or touching)	690	82.1
	Wash hands frequently and before meals to develop good personal hygiene habits	771	91.8

#### The high awareness rate of echinococcosis-related knowledge

The high awareness rate of echinococcosis-related knowledge of the residents in Xizang Autonomous Region was 66.1% (555/840). The qualified awareness rate of echinococcosis-related knowledge was not related to gender, age or occupation. With the improvement of the education level, the qualified awareness rate shows an upward trend (chi square for trend, χ^2^ = 21.23, *p*<0.05), as shown in Table 3.

**Table 3:** The qualified awareness rate of echinococcosis-related knowledge among residents of Xizang Autonomous Region, China, 2018 (*N* = 840)

Theme	Variables	Number of people surveyed	Number of qualified persons	The qualified awareness rate	χ^2^	*p*
Gender	Male	478	251	52.5	2.71	0.10
	Female	362	160	44.2		
Age (years)	≤20	38	7	18.4	7.84	0.25
	21~30	184	73	39.7		
	31~40	256	160	62.5		
	41~50	145	77	53.1		
	51~60	123	56	45.5		
	61~70	76	32	42.1		
	>70	18	6	33.3		
Education level	Illiterate	378	162	42.9	21.23^a^	0.00
	Primary	351	172	49.0		
	Secondary	59	38	64.4		
	University	52	39	75.0		
Principle occupation	Farmers	415	202	48.7	10.82	0.06
	Herdsmen	274	140	51.1		
	Other	50	20	40.0		
	Cadres or professional technicians	43	26	60.5		
	Workers	30	17	56.7		
	Students	28	6	21.4		

^a^chi-square for trend

### Investigation of echinococcosis-related attitudes

The high awareness rate of echinococcosis-related attitudes of the residents in Xizang Autonomous Region was 48.9% (411/840). The high awareness rate for males was higher than for females (χ^2^ = 5.70, *p*<0.05). The high awareness rate of echinococcosis-related attitudes among different age groups had a significant difference (χ^2^ = 48.43, *p*<0.05), and the 30–40 age group had the highest high awareness rate. With the improvement in the educational level, the high awareness rate shows an upward trend (chi-square for trend χ^2^ = 48.43, *p*<0.05). The high awareness rate of echinococcosis-related attitudes was not related to principle occupation (χ^2^ = 10.82, *p*>0.05), as shown in Table 4.

**Table 4:** The high awareness rate of echinococcosis-related attitudes among residents of Xizang Autonomous Region, China, 2018 (*N* = 840)

Theme	Variables	Number of people surveyed	Number of qualified persons	The qualified awareness rate	χ^2^	*p*
Gender	Male	478	327	68.4	5.70	0.02
	Female	362	228	63.0		
Age (years)	≤20	38	22	57.9	48.43	0.00
	21~30	184	125	67.9		
	31~40	256	173	67.6		
	41~50	145	97	66.9		
	51~60	123	82	66.7		
	61~70	76	46	60.5		
	>70	18	10	55.6		
Education level	Illiterate	378	232	61.4	23.34^a^	0.00
	Primary	351	243	69.2		
	Secondary	59	42	71.2		
	University	52	38	73.1		
Principle occupation	Farmers	415	288	69.4	7.15	0.21
	Herdsmen	274	167	60.9		
	Other	50	32	64.0		
	Cadres or professional technicians	43	32	74.4		
	Workers	30	19	63.3		
	Students	28	17	60.7		

^a^chi-square for trend

### Investigation of echinococcosis-related practices

The high awareness rate of echinococcosis-related practices of the residents in Xizang Autonomous Region was 47.9% (402/840). The development of echinococcosis-related practices was not related to gender and education level, instead they were related to age and principal occupation, as shown in Table 5.

**Table 5:** The high awareness rate of echinococcosis-related practices among residents of Xizang Autonomous Region, China, 2018 (*N* = 840)

Theme	Variables	Number of people surveyed	Number of qualified persons	The qualified awareness rate	χ^2^	*p*
Gender	Male	478	223	46.7	0.65	0.42
	Female	362	179	49.4		
Age (years)	≤20	38	11	28.9	52.72	0.00
	21~30	184	62	33.7		
	31~40	256	113	44.1		
	41~50	145	90	62.1		
	51~60	123	74	60.2		
	61~70	76	48	63.2		
	>70	18	4	22.2		
Education level	Illiterate	378	179	47.4	1.48	0.69
	Primary	351	165	47.0		
	Secondary	59	29	49.2		
	University	52	29	55.8		
Principle occupation	Farmers	415	197	47.5	20.63	0.00
	Herdsmen	274	124	45.3		
	Other	50	23	46.0		
	Cadres or professional technicians	43	30	69.8		
	Workers	30	17	56.7		
	Students	28	10	35.7		

## Discussion

Xizang is an autonomous region with a low socioeconomic status. It is sparsely populated with approximately 3.3 million inhabitants. Most inhabitants live in rural areas with poor sanitary facilities. A large proportion of inhabitants is affiliated with animal husbandry and agriculture. They come into close contact with animals and their living habits and daily practice are not strictly adhered to, hygiene principles, thus exposing them to a high risk of acquiring echinococcosis infection.

It is established that the transmission of echinococcosis is primarily related to educational level, human behavior, lifestyle, living habit and, environmental factors [24]. Health education can play a vital role in reducing transmission of echinococcosis to humans [25]. To prevent and control echinococcosis, it is necessary to draw awareness to improper behaviors, such as lifestyle and living habits that increase susceptibility to echinococcosis in the general population and guide the residents to establish good attitudes towards echinococcosis prevention and control, to change their bad behaviors. In underdeveloped or resource poor communities where education is inadequate and there are high levels of illiteracy, echinococcosis is often highly endemic [26].

This study revealed that the majority (86.8%) of the interviewed residents had a primary level of education or below that. During our investigation in the villages, feces shed by dogs were visible on the ground in most villages, providing evidence that a considerable proportion of dogs are allowed to roam free. This potentially endangers children who like to play and crawl on the ground, thereby increasing the oral-fecal route of transmission of echinoccocus [25].

A major challenge is to introduce educational materials on echinococcosis into targeted populations with low levels of literacy, and increase awareness of self-protection among the residents. Previous study has shown that due to a high proportion of illiteracy with few children going to school, education of dog owners and their children about echinococcosis control was only partly achieved on the Tibetan plateau [27]. Our study also confirmed that the education level of the participants affected their attitudes and practices towards echinococcosis. Data from our study showed the risk of echinococcosis was reduced with the increase in education level. Our study also revealed that only a small proportion of the interviewed residents are familiar with all the echinococcosis-related knowledge. A large proportion was not aware of the routes of echinococcosis infection in human or dogs. The lack of awareness of the echinococcosis infection routes led to ignorance the disease prevention. On the other hand, this strongly suggested that health education on echinococcosis in Xizang is not sufficient which worsen the epidemic of the disease.

Change in practice needs is much dependent on knowledge and attitudes. At present, both the high awareness rate of echinococcosis-related knowledge and attitudes of residents in Xizang Autonomous Region were low. Therefore, improving their echinococcosis-related knowledge and changing their attitudes might be the breakthrough points at this stage. Educational materials should be produced and distributed to the population. Considering the high level of illiteracy in the subjects, The education materials should be easy to understand and taught to the targeted population [24]. To highlight the intervention effect positively and create A strong social ethos that can encourage people to prevent and control echinococcosis is critically important for an effective intervention.

Our study only studied the relationship between demographic characteristics and knowledge, attitudes and practices. External factors related to the transmission of echinococcosis, such as environmental factors and family factors, need to be further explored.

## Conclusions

The epidemic of the disease has been largely resutantant from the lack of knowledge, awareness, and poor hygiene practice in local residences. Therefore, effective disease prevention education and awareness campaigns in community will be significantly helpful in prevention and control of echinococcosis. Improving the level of knowledge of echinococcosis is the basis for residents to prevent and control echinococcosis while changing attitudes is a key factor, and developing good practices represent effective means of achieving that goal.

## Supplementary information

**Additional file 1.** Questionnaire.

## Data Availability

All data and materials for this study shall be availed whenever requested by the editorial team, reviewers and other users. The data and materials can be requested from the corresponding author.

## Abbreviations

AE: Alveolar echinococcosis
CDC: Center for Disease Control and Prevention
CE: Cysticercosis echinococcosis
DALYs: Disability-adjusted life years
WHO: World Health Organization
YLLs: Years of life lost
