# Supplementary material for: Knowledge, attitudes and practices regarding echinococcosis in Xizang Autonomous Region, China
Source: BMC Public Health. 2020 Apr 15;20:483. doi: 10.1186/s12889-020-8314-8 (PMC7158018; doi:10.1186/s12889-020-8314-8)
Supplement: Supplementary file 1 — Additional file 1. Questionnaire. [file 12889_2020_8314_MOESM1_ESM.docx]

**编号**□□□□□□□□□（编码原则：县区3位+村庄3位+流水号3位）

Code□□□□□□□□□(Coding Principle: County 3 + Village 3 + Serial number 3)

2018年西藏自治区居民包虫病相关

知识、态度、行为（KAP）调查

Investigation on Knowledge, Attitude and Behavior (KAP) of echinococcosis among the Residents of Xizang Autonomous Region in 2018

**您好，我们是疾控中心工作人员，本调查主要了解您对包虫病的相关知识、态度、行为以及健康状况，您填写的信息我们不会公开，请按照您的真实情况认真回答，不要空项。**

**Hello, we are the staff of CDC. This survey mainly understands your knowledge, attitude, behavior and health status about echinococcosis. We will not disclose the information you fill in. Please answer carefully according to your actual situation and do not leave blanks.**

**一、基本情况Essential information**

1. 您的性别：①男 ②女

Your gender:①Male ②Female

2. 您的出生日期： 年 月 日或年龄 岁

Your date of birth: year month day or age

3. 民族：①藏族 ②汉族 ③回族 ④其他

Nation:①Tibetan ②Han ③Hui ④Others

4. 您的文化程度：①文盲 ②小学或初中 ③高中或中专 ④大专及以上

Your educational level: ①Illiteracy ②Primary or junior high school ③High school or technical secondary school ④.College or above

5. 您的职业：①干部或专业技术人员 ②工人 ③农民 ④牧民 ⑤学生 ⑥其他

Your occupation:①cadre or professional technician ②worker ③farmer ④herdsman ⑤student ⑥ other

6. 现住址： 县 村（学校）

Present address: County Village(School)

**二、包虫病相关知识调查**

**Investigation on knowledge of hydatidosis**

1. 您是否听说过包虫病（如肝包虫、肺包虫） □听过 □未听说过

Have you ever heard of echinococcosis(e.g. hepatic echinococcosis, pulmonary echinococcosis)

□Yes □No

2. 您觉得人是通过什么途径感染包虫病的？

How do you think people are infected with echinococcosis?

①含有虫卵的狗粪污染食物导致人、畜发病 □能 □不能 □不知道

Contaminated food by dog manure containing insect eggs can cause disease in humans or livestock □Yes □No □Don't know

②含有虫卵的狗粪污染水源导致人、畜发病 □能 □不能 □不知道

Contaminated water by dog manure containing insect eggs can cause disease in humans or livestock □Yes □No □Don't know

③人传染给人 □能 □不能 □不知道

Human-to-human transmission □Yes □No □Don't know

④除狗以外，狼和狐狸也可传播包虫病 □能 □不能 □不知道

Besides dogs, wolves and foxes can also transmit echinococcosis

□Yes □No □Don't know

4您觉得狗能通过下列途径感染吗？

Do you think dogs can be infected by the following ways?

①狗传染给狗 □能 □不能 □不知道

Can be transmitted by dog-to-dog □Yes □No □Don't know

②人传染给狗 □能 □不能 □不知道

Cannot be transmitted by human-to-dog □Yes □No □Don't know

③狗吃了生的带病的牛羊内脏而传染 □能 □不能 □不知道

Dogs are infected by eating the uncooked viscera of sick cattle and sheep

□Yes □No □Don't know

5. 您觉得包虫病能否导致下列危害？

Do you think echinococcosis can cause the following hazards?

①损害人体健康 □能 □不能 □不知道

Damage to human health □Yes □No □Don't know

②造成牛羊减产 □能 □不能 □不知道

Cause reduction of cattle and sheep production □Yes □No □Don't know

6. 您觉得下列途径可以预防包虫病吗？

Do you think the following ways can prevent echinococcosis?

①给狗驱虫 □能 □不能 □不知道

Expelling parasite for dogs □Yes □No □Don't know

②不用生的牲畜内脏喂狗 □能 □不能 □不知道

Cannot feed dogs with uncooked viscera of livestock □Yes □No □Don't know

③对狗粪进行处理（焚烧或深埋） □能 □不能 □不知道

Handle dog manure (be burned or deeply buried) □Yes □No □Don't know

④生活中不与狗密切接触（如亲吻或抚摸） □能 □不能 □不知道

Don’t be in close contact with dogs in life (such as kissing or touching)

□Yes □No □Don't know

⑤勤洗手、饭前洗手，养成良好个人卫生习惯 □能 □不能 □不知道

Wash hands frequently and before meals to develop good personal hygiene habits

□Yes □No □Don't know

**三、包虫病相关态度调查**

**Investigation of attitudes towards echinococcosis**

1. 您觉得您会得包虫病吗？ ①会 ②可能会 ③不会 ④不知道

Do you think you will get echinococcosis?

①Yes ②May ③No ④Don't know

2. 假如您被诊断为包虫病，您将 ①按照医嘱，坚持吃药 ②到寺庙向活佛寻求帮助

If you are diagnosed with echinococcosis, you will

①According to doctor's advice, insist on taking medicine

②Go to the temple and ask for help from the living Buddha

3. 如符合手术指征，是否愿意接受手术治疗 ① 是 ② 否

Are you willing to undergo surgical treatment if the indications are met?

①Yes ②No

4. 您是否支持捕杀流浪狗（无主犬）以控制包虫病的传播？①支持 ②不支持

Do you support killing stray dogs to control the spread of echinococcosis?

①Yes ②No

5. 假设您有一只狗，如果免费喂狗吃药驱虫，你愿意吗？ ①愿意 ②不愿意

Suppose you have a dog. Are you willing to expell parasite for your dog

by using daugs for free? ①Yes ②No

6. 您是否支政府持加强对居民养狗的管理（拴养、狗粪处理）？①支持 ②不支持

Do you support the government to strengthen the management of dog keeping (leashing, dog manure disposal)? ①Yes ②No

**四、包虫病相关行为调查**

**Investigation of echinococcosis-related Behavior**

1. 您吃饭前洗手吗? ①坚持洗手 ②有时洗手 ③偶尔洗手 ④很少洗手

Do you wash your hands before meals?

①Insist on washing ②Sometimes ③occasionally ④Seldom

2. 如果您有一只狗，你会选择哪种养狗方式 ①拴养 ②放养 ③白天拴养、晚上放养

If you had a dog, which way would you choose to raise it?

①Dog is tied ② Dog is untied ③Dog is tied in the daytime and released in the night

3.屠宰牛羊后的内脏怎么处理?（可多选）

How to deal with the viscera after slaughtering cattle or sheep?(Multiple choices)

①扔到垃圾堆 ②喂狗 ③埋掉 ④焚烧 ⑤其他（请描述）

①Throw it in the garbage dump ②Feeding dog ③Buried ④Burned ⑤Other

4. 日常生活中你有与狗玩耍吗？①经常 ②有时 ③偶尔 ④从不

Do you play with dogs in your daily life? ①Often ②Sometimes ③Occasionally

④Never

5. 您日常生活中是否手碰到过狗粪？ ①是 ②否

Have you ever touched dog manure in your daily life? ①Yes ②No

6. 家里的狗，是否驱虫？ ①家中无狗 ②已驱虫 ③未驱虫

Does the dog of yours expelled parasite? ①No dog ②Yes ③No

**五、包虫病宣传方面的情况**

**Information on echinococcosis propaganda**

1. 您是否见过社区组织的包虫病防控知识宣传活动？ ①是 ②否（请跳至第3题）

Have you seen the echinococcosis prevention and control knowledge campaign organized by the community? ①Yes ②No(Please skip to Question 3.)

2. 如果是，是什么形式的？If yes,what form is it?

①社区医生宣传 ②电视、广播、网络、微信 ③报刊、杂志 ④板报、宣传栏、海报、宣传册（单）⑤家人或朋友 ⑥其他（请列出）

①Community Doctor Advocacy ②Television, Radio, Network, Wechat

③Newspapers and magazines ④Boards, billboards, posters, brochures (sheets)

⑤Family or friends ⑥Other(Please list)

3. 您的包虫病防病知识主要来源于：

Your knowledge of echinococcosis prevention mainly comes from:

①社区医生宣传 ②电视、广播、网络、微信 ③报刊、杂志 ④板报、宣传栏、海报、宣传册（单）⑤家人或朋友 ⑥其他（请列出）

①Community Doctor Advocacy ②Television, Radio, Network, Wechat

③Newspapers and magazines ④Boards, billboards, posters, brochures (sheets)

⑤Family or friends ⑥Other(Please list)

4.您希望通过什么途径获取包虫病方面的知识和信息？

How would you like to acquire knowledge and information about hydatidosis?

①社区医生宣传 ②电视、广播、网络、微信 ③报刊、杂志 ④板报、宣传栏、海报、宣传册（单）⑤家人或朋友 ⑥其他（请列出）

①Community Doctor Advocacy ②Television, Radio, Network, Wechat

③Newspapers and magazines ④Boards, billboards, posters, brochures (sheets)

⑤Family or friends ⑥Other(Please list)

**调查人员： 调查日期：**

**Investigator： Date of survey：**
